# The Regulation of Inflammatory Pathways and Infectious Disease of the Cervix by Seminal Fluid

**DOI:** 10.1155/2014/748740

**Published:** 2014-08-11

**Authors:** Anthonio Adefuye, Arieh Anthony Katz, Kurt Jason Sales

**Affiliations:** MRC/UCT Receptor Biology Research Unit, Institute of Infectious Disease and Molecular Medicine and Division of Medical Biochemistry, Faculty of Health Sciences, University of Cape Town, Room No. 2.02 Wernher and Beit North, Observatory 7925, South Africa

## Abstract

The connection between human papillomavirus (HPV) infection and the consequent sequelae which establishes cervical neoplastic transformation and invasive cervical cancer has redefined many aspects of cervical cancer research. However there is still much that we do not know. In particular, the impact of external factors, like seminal fluid in sexually active women, on pathways that regulate cervical inflammation and tumorigenesis, have yet to be fully understood. HPV infection is regarded as the initiating noninflammatory cause of the disease; however emerging evidence points to resident HPV infections as drivers of inflammatory pathways that play important roles in tumorigenesis as well as in the susceptibility to other infections such as human immunodeficiency virus (HIV) infection. Moreover there is emerging evidence to support a role for seminal fluid, in particular, the inflammatory bioactive lipids, and prostaglandins which are present in vast quantities in seminal fluid in regulating pathways that can exacerbate inflammation of the cervix, speed up tumorigenesis, and enhance susceptibility to HIV infection. This review will highlight some of our current knowledge of the role of seminal fluid as a potent driver of inflammatory and tumorigenic pathways in the cervix and will provide some evidence to propose a role for seminal plasma prostaglandins in HIV infection and AIDS-related cancer.

## 1. Introduction

Cervical cancer is a malignant neoplasm arising from the cervical squamous or columnar and glandular epithelial cells that line the cervix-uteri in women. Neoplastic transformation of these cells is initiated by infection of the cervical epithelium with high risk oncogenic types of human papillomavirus (HPV) and is largely regarded as an infectious disease [1, 2]. Around 180 different types of HPV have been described to date [3]. Of these, around 40 types are known to infect the anogenital tract, giving rise to genital warts, condylomata or cancers, and their precursor lesions [4]. The primary route for genital infection is via exposure of the mucosa (vaginal, oral, or anal) to HPV present in bodily fluids (mainly seminal fluid, but can also be passed on by saliva) or via skin to skin contact and transmission during sexual intercourse [4].

HPV infects the basal keratinocyte [4]. After infection the cell divides and the life cycle of the virus closely follows that of the keratinocyte as it differentiates and matures. Early viral genes, such as the E6 and E7 oncogene, are expressed in the undifferentiated basal and parabasal layers, whilst expression of the late genes and viral DNA replication occurs in the upper more differentiated granular or cornified layers of the epithelium [4, 5]. The virus is a molecular “hitchhiker” completely lacking any ability to facilitate its own replication and hence is solely reliant on the cellular machinery of its host for propagation. As the cell differentiates, the viral capsid forms and the resultant virion can be released at the cell surface to infect other host cells [4–6] or to be passed on during sexual intercourse to infect another individual. During the initial phase of infection, the HPV E6 and E7 oncogenes are transcribed and translated and it is the E6 and E7 oncoproteins that do most of the cancer-transformation of the infected cell. Recent studies have now shown that HPV mediated transformation occurs by chromosomal alterations, due in part to the actions of the HPV E6 and E7 oncogene, which induce epigenetic changes and changes in miRNA expression to facilitate cellular immortality and neoplastic transformation [7–11].

HPV infections are nonlytic and as such, the host does not mount an inflammatory response at the onset of infection. However, it is emerging that resident HPV oncogenes can regulate cellular effectors, including miRNAs and genes involved in tissue remodeling events in neoplastic cervical epithelial cells, which are critical regulators of the inflammatory processes in cancerous tissue [12, 13].

Emerging evidence points to a role for resident HPV oncogenes in driving tumor-associated inflammation in immortalized cells by inducing the inflammatory cyclooxygenase- (COX-) prostaglandin (PG) axis and elevating cytokine networks [14–16]. Two isoforms of cyclooxygenase enzymes have been described in humans, namely, COX-1 and COX-2, which catalyze the rate limiting conversion of arachidonic acid, derived by de-esterification of plasma membrane phospholipids or dietary polyunsaturated fatty acids, to eicosanoids (prostaglandins, thromboxanes, and prostacyclins) [17]. Many chronic inflammatory diseases and cancers are all associated with upregulation in COX enzyme expression and aberrant biosynthesis of proinflammatory eicosanoids [18, 19]. Elevated signaling of prostaglandins has been observed in uterine-cervical cancers and is considered a key modulator of tumor progression [19–22].

Prostaglandins mediate their effects via coupling to specific prostanoid G protein-coupled receptors (GPCRs) [23]. In the past two decades, many studies have highlighted that prostaglandins, produced as a consequence of elevated COX enzyme expression, can act via these prostanoid GPCRs to promote extensive tissue remodeling within tumours by evoking all the classical hallmarks of cancer, namely, cellular proliferation, angiogenesis, inhibition of apoptosis, and alteration in vascular permeability, to allow immune cell extravasation from the vasculature and inflammation [19, 24]. These hallmarks of cancer in cervical cancer cells have now all been shown to be driven by HPV oncogenes via the induction of potent proinflammatory pathways—in particular, by inducing expression of the immediate early oncogene COX-2 and expression of the E-series prostaglandin receptors (PTGER) such as PTGER2 and PTGER4 [14–16]. In addition to the regulation of tumorigenic pathways by endogenous prostaglandins, produced in cervical cells after HPV infection and neoplastic transformation, the cervix (and vagina) can also be regulated by seminal fluid and in particular seminal plasma prostaglandins, like PGE_2_ [25–27].

## 2. The Role of Seminal Fluid in Regulating Cervical Inflammation and Tumorigenesis

The male ejaculate is a complex organic fluid comprising of secretions of multiple bioactive molecules derived from the Cowper's and Littre's glands (5%), prostate (15–30%), and the seminal vesicles (65–70%) [28]. Secretions from each of these reproductive organs are biochemically distinct and on mixing, as occurs at ejaculation, give rise to the complex biochemical nature of the seminal plasma (SP; also called seminal fluid), the main constituent of the ejaculate. The spermatozoa during ejaculation are bathed in this fluid, which has conventionally been viewed as a nutritive, protective, and transport medium for the mammalian spermatozoa [29]. Semen deposition into the female reproductive tract results in modulated immunity and introduction of an inflammatory response in the mucosa, which promotes conditions for favorable conception and pregnancy [29, 30]. In rodents and humans, studies have shown that SP contains signaling molecules which can bind to cognate epithelial receptors in the female reproductive tract to trigger inflammatory gene expression, modifications in cellular structure, and tissue remodeling events in a sequence that closely resembles an inflammatory response [29].

In mammals, the most striking physiological effects of this inflammatory response can be observed after insemination, where a prompt and dramatic influx of inflammatory cells can be observed to infiltrate the site of semen deposition [29]. These cellular changes have been studied in detail in rodents, where a surge in inflammatory cytokines and chemokines can be seen in cervical and uterine epithelial cells after administration of seminal plasma [31–33]. These proinflammatory mediators stimulate the extravasation and infiltration of cervical subepithelial stroma by immune cells. Similarly, in humans coitus elicits immune cell (neutrophils, macrophages, dendritic cells, and lymphocytes) recruitment into the superficial epithelial and deep stroma tissues of the female reproductive tract [34].

In sexually active women, the degree at which SP normally activates the secretion of these proinflammatory components in any compartment of the female reproductive tract is poorly understood. However, exposure of the cervix to seminal plasma during coitus has been shown to elicit substantial changes in the leukocyte populations within the cervix, initiating a reaction reminiscent of the inflammatory response with effects that penetrate through the stratified epithelial layer and deep into the stroma of the ectocervix [35]. The role of seminal plasma in these studies in mediating leukocyte influx in the cervix was confirmed in control subjects where an absence of inflammatory response was seen with condom-protected coitus or in women abstaining from coitus [35].

**Figure 1:**
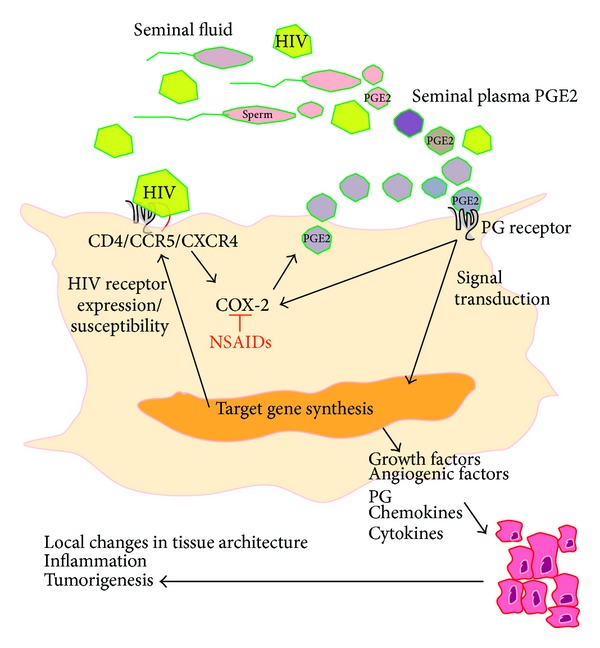
A cartoon highlighting the role of seminal plasma and seminal plasma prostaglandins (PG) such as PGE_2_ in regulating inflammatory and tumorigenic pathways in cervical cancer cells. Seminal plasma PGE_2_, or PGE_2_ endogenously produced by COX enzymes in response to inflammation or HIV infection via the induction of COX-2, binds to PG receptors on the surface of the cell to activate intracellular signal transduction pathways and target gene transcription and biosynthesis. Target genes which are known to be regulated in this manner include HIV chemokine receptors like CXCR4 as well as growth factors, angiogenic factors, chemokines, and cytokines. These latter factors all act in an autocrine/paracrine manner in the tumour microenvironment to facilitate local changes in tissue architecture, inflammation and enhance tumorigenesis. Elevated expression of HIV receptors could enhance virus entry into cells and enhance susceptibility to infection. HIV is also known to enhance inflammation by inducing COX-2 expression. The activity of COX-2 and subsequent inflammation can be inhibited by nonsteroidal anti-inflammatory drugs (NSAIDs). The activity of specific PG receptors can also be inhibited with selective receptor antagonists to inhibit subsequent activation of signal transduction pathways.

Seminal plasma is thought to control the influx of immune cells and expand inducible regulatory T cell population to produce immune tolerance for conception [35–37] via the activation of proinflammatory cytokine/chemokine networks [38]. Under pathological conditions or in women with neoplastic cervical lesions—post-HPV infection—the inflammatory response initiated by SP could be exaggerated and could drive pathways which regulate tumorigenesis. Indeed,* in vitro* studies have shown that SP induces the expression of inflammatory COX-1 and COX-2, interleukin (IL)-6, IL-11, and chemokines CXCL1 and CXCL8 in cervical adenocarcinoma cells [26, 39]* in vitro* and in animal models* in vivo* to regulate blood vessel recruitment and angiogenesis (Figure 1) [26, 39]. The induced expression of these effector molecules in neoplastic cervical epithelial cells by direct stimulation with seminal plasma suggests that repeated exposure of the cervix to seminal fluid in the absence of barrier contraception could exacerbate tumour-associated inflammation and could enhance disease progression (Figure 1).

In addition to direct exposure of the epithelial layer surrounding the anterior and posterior vaginal fornix, ecto- and endocervix to seminal fluid during intercourse, direct absorption of components of seminal plasma can enter into the endometrial or peritoneal bed. The intravaginal absorption of male hormones has been described [40], and vaginal absorption of molecules is widely regarded as a potential means of drug delivery in women. Absorption may occur as a result of hematogenous dissemination or direct tissue perfusion through the anterior or posterior vaginal fornix [41]. Although it is likely that the concentrations of inflammatory mediators in SP in the recipient woman after intercourse would be orders of magnitude lower than that found in semen, concentrations of inflammatory mediators would nevertheless be greater in sexually active women and could impact* in situ* on inflammation and tumour growth. Indeed, prostaglandins, especially PGE_2,_ are present in seminal plasma in orders of magnitude greater than at the site of infection or endogenously regulated inflammation [42]. PGE_2_ has recently been described as the main constituent of seminal fluid responsible for regulating inflammatory gene expression in vaginal epithelial cells [27]. Since expression of the PGE_2_ receptors is elevated in cervical cancers [21, 22], it is likely that seminal plasma (and in particular the PGE_2_ present in seminal plasma) can regulate inflammatory and tumorigenic pathways directly via paracrine interactions with their cognate GPCRs in the cervix to regulate many of the hallmarks of tumorigenesis, including angiogenesis (Figure 1) [43, 44], cellular proliferation, and immune cell regulation.

## 3. The Role of Seminal Plasma in Regulating HIV Infection and Its Potential Contribution to AIDS-Related Cervical Cancer

The origins of human immunodeficiency virus (HIV) are thought to predate the first half of the 20th century, where zoonotic transmission of simian immunodeficiency virus (SIV), found in chimpanzees, occurred, giving rise to HIV, as we know it today [45]. To date, HIV has infected more than 60 million people and caused more than 25 million deaths [46]. Current data estimate that approximately 35 million people currently live with HIV worldwide [46]. In 2012 alone there were around 2.3 million new infections and 1.6 million deaths due to HIV/AIDS worldwide [46]. These figures highlight that 30 years on, after the first reported cases of HIV/AIDS, HIV still remains a major global health pandemic and one of the leading causes of death and disease in women of reproductive age [47]. Morbidity is driven by depletion of the immune system. HIV attacks the immune system by targeting CD4^+^ cells and increases the risk of acquiring opportunistic infections. In 1993, cervical cancer was classified as an AIDS-defining disease in women infected with HIV [48], highlighting HIV as an important risk factor/cofactor in the development of invasive cervical cancer.

It has been well documented that unprotected sexual intercourse is the major route of HIV infection with infected seminal plasma being the major transmission vector [49–51]. After deposition of semen into the female genital tract, dramatic tissue remodeling events occur [29, 52, 53]. Although these events are thought to promote fertilization and pregnancy, the alterations to the local cellular environment could also enhance HIV susceptibility. Some of the alterations in the local environment which could impact on HIV susceptibility include immune cell recruitment and activation; alterations in epithelial integrity; alterations in the mucous barrier in the genital tract; alterations in the levels of naturally occurring flora and antimicrobials in the genital tract; and alterations in the balance of inflammatory/anti-inflammatory mediators in the genital mucosa [54].

Once HIV has been deposited within the genital tract, infectious virus must cross the mucosal epithelium to interact with macrophages, CD4^+^ T lymphocytes, and dendritic cells (DCs). Transmission occurs via transcytosis of virions through the genital epithelium [50, 55] or through exposed genital lesions in the mucosa [56–58] or microabrasions in the vagina [59].

HIV infects cells via receptors on the host cell surface. Initially, the virus attaches to the surface of the host cells. This can occur via heparin sulphate proteoglycans [60]. The initial step in membrane fusion begins with binding of the viral envelope protein (Env, consisting of a trimer of gp120-gp41 heterodimers) to the CD4 cell surface protein and a chemokine coreceptor present on the host cell [61]. Most HIV-1 variants use CCR5 and CXCR4 as the main coreceptor* in vivo*; however up to 12 other chemokine coreceptors for HIV infection have been identified* in vitro* [62, 63].

The cervicovaginal epithelium displays heterogeneous tissue architecture, comprising a multilayered stratified squamous epithelium at the vaginal-ectocervical interface, whilst the endocervix is lined with a single layered columnar epithelium. The epithelial surface expresses all the receptors necessary for HIV infection including CD4, CCR5, CXCR4 [64, 65], and various other G protein-coupled coreceptors (GPCRs) known to mediate entry of HIV into cells (including CCR2b, CXCR6, and GPR1) [63, 66], indicating a vast area of the female genital tract that can potentially be infected. Furthermore the transformation zone (the interface between the squamous ectocervix and columnar endocervical canal) is thought to be particularly susceptible to HIV infection as it has an enhanced population of CD4^+^ T cells [55, 67]. Recent studies have highlighted that cervical epithelium can become productively infected and behave as viral reservoirs, which sequester virus and facilitate introduction of virus to leukocytes present in the submucosa [68, 69].

## 4. The Role of Semen in Modulating HIV Infectivity

Beyond its role as a carrier for delivery of HIV during receptive vaginal or anal intercourse, semen has been suggested to play a more major role in HIV transmission. In addition to local changes in tissue architecture, inflammatory cell recruitment, and secretion of inflammatory mediators in the female genital tract, semen may facilitate or inhibit mucosal HIV infection via multiple mechanisms to impact on susceptibility.

One mechanism by which semen could enhance sexual transmission of HIV was demonstrated by Munch and colleagues in 2007 [70]. To identify natural agents that might play a role in sexual transmission of HIV, Munch and colleagues developed a complex peptide/protein library derived from human seminal plasma which was screened for novel inhibitors and enhancers of HIV infection. They found that naturally occurring fragments of the prostatic acidic phosphatase (PAP) dramatically enhance HIV infection [70]. Functional and structural analyses showed that PAP forms amyloid fibrils, termed semen-derived enhancer of virus infection (SEVI), which markedly increased HIV infection [70]. SEVI capture HIV and promote the attachment and fusion of the virus to the cell surface to increase infection of both R5 and X4 tropic HIV-1 to peripheral blood mononuclear cells (PBMCs), DCs, and macrophages* in vitro*. In an* in vivo* experiment to demonstrate the enhancing effect mediated by SEVI, using hCD4/hCCR5-transgenic rats challenged with HIV-1 or SEVI-treated HIV-1 by tail vein injection, it was discovered that pretreatment of HIV with SEVI resulted in a 5-fold increase in the number of HIV cDNA copies found in the splenocyte extracts from infected rats [70]. The increase in HIV infectivity when treated with SEVI* in vitro* suggests that it might favor sexual transmission of HIV. This, however, has been shown to be highly dependent on the individual semen donor and correlates with the level of SEVI [71].

Another study highlighting a role for semen in enhancing HIV-1 infections has been reported by Bouhlal and colleagues (2002) [72]. These authors showed that opsonization of HIV-1 by semen complement fragments enhances infection in human epithelial cells. In this study, it was demonstrated that both R5- and X4-tropic viruses could infect epithelial cells and that infection of cells was enhanced 2-fold when virus was added to semen prior to incubation with the epithelial cells and that this effect was complement dependent [72]. There is also evidence that semen can raise the pH of the vaginal fluid from a normally acidic pH to a neutral pH 7. This natural buffering capacity of semen might further enhance HIV stability and create an environment conducive to infection [73, 74]. Other proposed mechanisms of semen-mediated HIV transmission include the role of spermatozoa in efficient transmission of HIV to dendritic cells [75], semen-mediated inflammation [76], semen-mediated immunomodulation, and suppression of both innate and adaptive immune response against HIV [77], and more recently semen lactoferrin has been shown to promote expression of CCL20 in epithelial cells to potentially enhance HIV transmission [78].

Although semen has been shown to be permissive to infection of certain cell types there is also evidence that certain constituents of seminal plasma can impede HIV infectivity. For example, mucin-6 can prevent the capture of HIV-1 by dendritic cells (DC) such as DC-SIGN (DC-specific-intracellular-adhesion-molecule-3-grabbing-nonintegrin) [79, 80]. Productive infection of certain DC* in vivo* is still a matter of debate; however, they are important for early HIV pathogenesis and transfer of virus to CD4^+^ T cells [81, 82]. In addition cationic polypeptides [83] contained in seminal plasma and reactive oxygen species produced by leukocytes and spermatozoa are known to provide antiviral activity against HIV [84]. More research is needed to elucidate the collective effects of all these effectors in semen in modulating the cervico-vaginal environment after intercourse and the impact on HIV susceptibility in women.

## 5. The Role of Semen in Regulation of Cell Surface Receptors and Signaling

Constituents of seminal plasma, including TGF*β*, EGF, prostaglandins, and soluble complement components could directly regulate HIV susceptibility via the interaction with or modulation of expression of cell surface receptors present on mucosal epithelial and immune cells [19, 25, 29, 52, 85]. For example, Bouhlal and colleagues showed that complement activation in seminal fluid generates C3 cleavage and augments HIV-1 infection in epithelial cells via a complement receptor type 3- (CR3-) mediated mechanism [72]. CR3 is expressed on the apical surface of epithelial cells including cells of the ecto- and endocervix, endometrium, and fallopian tube [86]. This suggests that opsonization of HIV with complement is important in the early events surrounding mucosal transmission of HIV [87]. Furthermore as highlighted earlier, the epithelial surface expresses all the receptors necessary for HIV infection including CD4, CCR5, CXCR4 [64, 65], and various other G protein-coupled coreceptors (GPCRs) known to mediate entry of HIV into cells (including CCR2b, CXCR6, and GPR1) [63, 66]. Some of these receptors display cyclical variations during the menstrual cycle indicating that susceptibility of cells could be different in women, depending on the phase of the menstrual cycle [65]. In women with genital tract pathologies, and cervical cancers in particular, expression of chemokine receptors such as CXCR4 and CCR5 is elevated [88, 89]. Chemokine receptors like CXCR4 are known to play a role in lymph node metastasis during advanced-stage disease [89]; however their elevated expression in cervical cancers could potentially be hijacked by HIV for entry. Furthermore, CXCR4 expression can be regulated by HPV oncogenes [90] and prostaglandins (PGE_2_) [91] in the female genital tract (Figure 1) and could potentially be enhanced following sexual exposure to semen and seminal plasma PGE_2_. Indeed, seminal plasma has been shown to increase CCR5 expression in target lymphocytes [92], and seminal plasma and PGE_2_ have been shown to increase CCR5 expression in cervical epithelial cells via induction of the PGE_2_ receptor signalling pathway [88], indicating the potential for enhancing the preferential transmission of R5 tropic HIV. These observations suggest that HPV infection, inflammatory mediators, and semen can drive expression of HIV coreceptors on cervical epithelial cells. Elevated HIV receptors, which can themselves be regulated by seminal plasma prostaglandins [88], could then enhance the susceptibility of these women to HIV infection.

As highlighted earlier, cervical cancer is an AIDS-related disease. Recently Fitzgerald and colleagues have demonstrated that HIV infection in women with HPV positive cervical cancers increases COX-2 expression and systemic PGE_2_ levels [93]. In cervical cancers, expression of the PGE_2_ receptors is elevated in cervical epithelial cells [21]. In the case of women with dual HPV and HIV infection, where PGE_2_ receptor expression is elevated together with elevated circulating PGE_2_ concentrations, the enhanced ligand-receptor signalling mediated by PGE_2_ could potentially exacerbate inflammation and drive tumour progression and morbidity. These data provide some compelling evidence into a possible mechanism whereby HIV infection can increase AIDS-related cervical cancer progression in women with cervical cancer.

## 6. Conclusions

There is significant evidence that the cervicovaginal microenvironment is modified during sexual intercourse by semen to enhance conception and pregnancy via the remodeling of multiple tissue compartments. Opportunistic infections and HIV present during intercourse can hijack the pathways mediating this response to seminal fluid and can be enhanced by factors present in seminal fluid. As demonstrated schematically in Figure 1, there is further evidence for a role for seminal plasma and seminal plasma prostaglandins, like PGE_2_, in regulating inflammation and pathways which regulate tumor progression in cervical cancers. Furthermore, there is evidence that seminal plasma and PGE_2_ can increase susceptibility to HIV infection by modulating the local inflammatory response in the cervix and elevating expression of HIV receptors in cervical epithelial cells and lymphocytes. Once infected, HIV can potentially enhance tumorigenesis by increasing systemic PGE_2_, which has been shown to promote tumour progression. Nonsteroidal anti-inflammatory drugs (NSAIDs) that inhibit the synthesis of PGE_2_, or drugs that inhibit the actions of PGE_2_, like specific PGE_2_ receptor antagonists, have demonstrated dramatic anti-inflammatory and antineoplastic activity for a variety of cancers [94, 95] by inhibition of the COX-prostaglandin pathway. These compounds may prove useful in reducing inflammation and the progression of AIDS-related cervical cancer in women and may also prevent the semen-induced upregulation in cellular machinery that could enhance HIV infection. More research is needed to determine the potential effectiveness of such interventions in women's health.
